# Research on the Conductivity-Based Detection Principles of Bubbles in Two-Phase Flows and the Design of a Bubble Sensor for CBM Wells

**DOI:** 10.3390/s16091520

**Published:** 2016-09-17

**Authors:** Chuan Wu, Guojun Wen, Lei Han, Xiaoming Wu

**Affiliations:** 1 Faculty of Engineering, China University of Geosciences (Wuhan), Wuhan 430074, China; wuchuancug@126.com (C.W.); weiliu181@126.com (X.W.); 2 Faculty of Mechanical and Electronic Information, China University of Geosciences (Wuhan), Wuhan 430074, China; hanleicug@126.com

**Keywords:** bubble sensor, gas-liquid two-phase flow, two-phase flow bubble, conductivity, coalbed methane (CBM)

## Abstract

The parameters of gas-liquid two-phase flow bubbles in field coalbed methane (CBM) wells are of great significance for analyzing coalbed methane output, judging faults in CBM wells, and developing gas drainage and extraction processes, which stimulates an urgent need for detecting bubble parameters for CBM wells in the field. However, existing bubble detectors cannot meet the requirements of the working environments of CBM wells. Therefore, this paper reports findings on the principles of measuring the flow pattern, velocity, and volume of two-phase flow bubbles based on conductivity, from which a new bubble sensor was designed. The structural parameters and other parameters of the sensor were then computed, the “water film phenomenon” produced by the sensor was analyzed, and the appropriate materials for making the sensor were tested and selected. After the sensor was successfully devised, laboratory tests and field tests were performed, and the test results indicated that the sensor was highly reliable and could detect the flow patterns of two-phase flows, as well as the quantities, velocities, and volumes of bubbles. With a velocity measurement error of ±5% and a volume measurement error of ±7%, the sensor can meet the requirements of field use. Finally, the characteristics and deficiencies of the bubble sensor are summarized based on an analysis of the measurement errors and a comparison of existing bubble-measuring devices and the designed sensor.

## 1. Introduction

China has rich reserves of coalbed methane (CBM). Coalbed methane buried below 2000 m covers an area of 36.81 trillion m^3^, accounts for approximately 15.3% of the world’s coalbed methane reserves, and ranks as the third largest in the world [1,2]. To make full use of coalbed methane resources, in recent years the Chinese government has stepped up the research and development of coalbed methane extraction technologies and drilled several test wells to explore a set of coalbed methane extraction techniques.

Due to the natural fracture structure of coalbeds, CBM wells need drainage and depressurization, and in that process groundwater and coalbed methane are produced from the wellbore, which results in the occurrence of gas-liquid two-phase flows in the wellbores. For vertical CBM wells, the extraction of coalbed methane is controlled by detecting and adjusting the working parameters of gas-liquid two-phase flows in the wellbores. Based on this, domestic and foreign scholars have conducted extensive research on the related characteristic parameters of two-phase flows, and the detection and research of bubble parameters (including the ascending velocities and volumes of bubbles) is the highest priority in studying the parameters of two-phase flows [3,4].

Theoretical research on two-phase flow bubbles of coalbed methane based on laboratory simulation tests is the focus of current research. The existing data show that the theoretical forecasting research results of different scholars differ greatly, have significant errors, and are inconsistent with actual production [5,6,7]. Therefore, a more direct, reliable, and accurate method, namely the real-time detection of bubble parameters during the operation of CBM wells, can be adopted. Further studies can be carried out according to the actual detected results. 

The existing detection methods for the bubble parameters of two-phase flows cannot be applied to the working conditions of CBM wells. These methods include bubble detection methods based on light scattering imaging [8], the machine vision principle [9], the signal attenuation principle [10,11], tomographic imaging technology [12,13], etc.

### 1.1. The Bubble Detection Method Based on Light Scattering Imaging

The laser beam generated by the laser irradiates the water. When the water contains bubbles, the incoming beam will deviate from the original position and scatter in other directions. Due to the different scattered light intensities of different forms of bubbles in different directions, the bubble scattered light in a specific direction can be imaged using a Charge Coupled Device (CCD) camera. After image processing and theoretical calculations, the volume, velocities, and distribution of the bubbles can be obtained.

This detection method is inapplicable to the working conditions of CBM wells for the following reasons.

The sizes of the laser and CCD camera are larger than the annular space size (approximately 25 mm) in CBM wells, so the well space fails to meet the installation requirements.

The sealing performances of existing lasers and CCD cameras cannot meet the requirements (liquid pressure exists in the well, and the pressure of a 1000 m deep CBM well is generally approximately 10 MPa).

No light source exists in the well, so if a CCD camera is used, additional continuous light must be provided, or the flashlight of a CCD camera must be powered on, which will increase the complexity of the detection system and reduce its reliability while also placing greater demands on the power supply system.

### 1.2. The Bubble Detection Method Based on the Machine Vision Principle

When the water contains bubbles, a high-speed camera is used to image the water continuously, and the photos are then analyzed and computed using image processing technology, thus obtaining the volume, velocities, and distribution of the bubbles.

This detection method is inapplicable to the working conditions of CBM wells for the following reasons. 

The bubble detection method based on the machine vision principle mainly comprises a high-speed camera that is similar to a CCD camera. Therefore, this method cannot meet the operating requirements of CBM wells for the same reason as the above light-scattering imaging principle.

### 1.3. The Bubble Detection Method Based on the Signal Attenuation Principle

An electromagnetic wave (or ultrasonic) signal generator sends an electromagnetic wave (or ultrasonic) signal of a certain power according to a set frequency. The transmitted electromagnetic wave (or ultrasonic) signal is received by an electromagnetic wave (or ultrasonic) signal receiver after passing the two-phase flow. By virtue of the different attenuation amounts of the electromagnetic wave (or ultrasonic) signal passing the gas-phase and liquid-phase, the flow pattern, volume, and velocity of the two-phase flow can be acquired using the statistics of the amount and time of the electromagnetic wave (or ultrasonic) signal attenuation using computer software.

This detection method cannot be applied under the working conditions of CBM wells for the following reasons. 

In the use of a bubble detector based on the signal attenuation principle, it must be ensured that the electromagnetic wave (or ultrasonic) signal generator and electromagnetic wave (or ultrasonic) signal receiver are accurately installed at the well bottom. However, the annular space size in CBM wells is generally approximately 25 mm, which is too small. In this case, it cannot be ensured that the electromagnetic wave (or ultrasonic) signal generator and electromagnetic wave (or ultrasonic) signal receiver are installed with high alignment accuracy. Moreover, the sizes and sealing performances of the electromagnetic wave (or ultrasonic) signal generator and electromagnetic wave (or ultrasonic) signal receiver also cannot meet the operating requirements in CBM wells. Thus, this detection method is inapplicable.

### 1.4. The Bubble Detection Method Based on Tomographic Imaging Technology

The method is usually realized by adding a drive signal to the two-phase fluid. Because the attenuation amounts of the gas-phase and liquid-phase are different, the sectional phase distribution diagrams at different angles and directions can be obtained by analyzing the different signal attenuation. According to the obtained phase distribution, the flow pattern of the two-phase flow, sectional phase flow rate, and approximate diameter of the bubbles on a section can be obtained. This method includes, for example, the electrical capacitance tomography, electric resistance tomography, X-ray image formation, and γ-ray imaging.

This detection method cannot be applied under the working conditions of CBM wells for the following reasons. 

The bubble detection method based on tomographic imaging technology cannot be applied under the working conditions of CBM wells because this method requires a higher installation precision. The sealing performances and size of this method are also inapplicable to the working conditions of CBM wells. In addition, the method requires real-time imaging that requires extremely high data sampling frequency. According to signal transmission line theory, a high-frequency signal will attenuate when transmitted to the surface through the cable in the well, leading to signal distortion. Thus, this detection method is inapplicable. 

### 1.5. Other Detection Methods

In addition to the above detection methods, there are many other kinds of detection methods such as the conductivity detection method [14], optical fiber detection method [15], acoustic detection method [16], light polarization detection method [17], etc.

The methods are inapplicable under the working conditions of CBM wells because of their installation sizes and precision, sealing performances, and data transmission speeds, for example.

Overall, the common bubble detection methods and measuring devices cannot meet the detection requirements in the working conditions of CBM wells, primarily due to limitations of their measuring principles, installation sizes, installation accuracy, and sealing performances, among other factors. Nevertheless, these methods can be used to detect the basic forms of bubbles, including their quantity, velocity, and volume, and the flow patterns of two-phase flows.

This paper therefore reports on the design of a new bubble sensor that can detect the flow patterns of two-phase flows and bubble velocities and volumes in real time based on conductivity detection principles.

## 2. Research on Conductivity Detection Principles of Two-Phase Flow Bubbles in Wells

According to the differences between the conductibility of liquids and bubbles, the conductivity detection principles regarding the passing, quantities, flow patterns, velocities, volumes, and other parameters of two-phase flow bubbles wells are put forward.

### 2.1. Liquid Conductivity 

Liquid conductivity is a parameter used for measuring the electrical conductivity of a liquid. To measure the electrical conductivity of a liquid, the first step is to insert two inert electrodes into a liquid sample, and the next step is to energize one of the electrodes with a DC positive voltage and ground the other electrode. The calculation equation of liquid conductivity at constant temperature is then

(1) $$K=\frac{L_{d}}{AR_{d}},$$

where *K* is the liquid conductivity, *L_d_* is the distance between the two electrodes, *A* is the sectional area of each electrode, and *R_d_* is the resistance between the two electrodes.

In Equation (1), *L* and *A* are associated with the shape and size of an electrode, and *L/A* is constant after the electrodes are completed. *R_d_* is related to the temperature, and as long as the resistance *R_d_* between the two electrodes is measured under a particular temperature, the electrical conductivity of the solution can therefore be obtained.

### 2.2. Detection Principles of Bubble Passing

**Figure 1:**
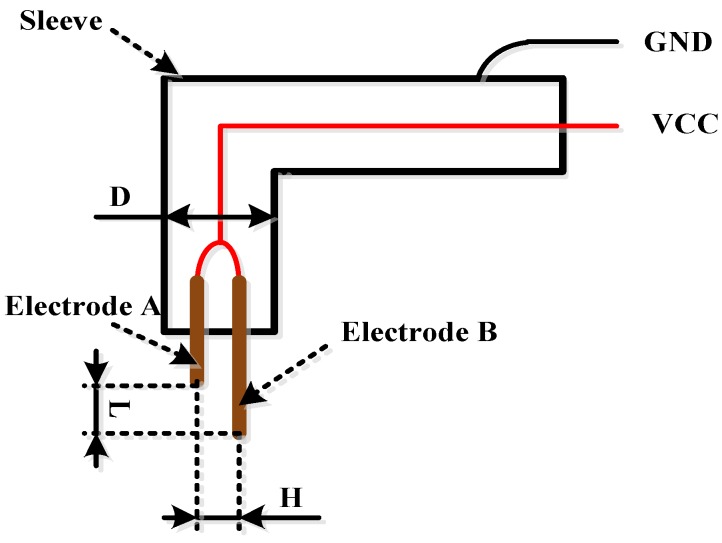
Schematic diagram of the structure of the bubble detection sensor.

Figure 1 shows a schematic diagram of the structure of the bubble detection sensor. The bubble sensor comprises a sleeve, Electrode A, and Electrode B. The sleeve material is stainless steel, and that of the electrodes is conductive metal. The diameter of each electrode is 0.3 mm. The two electrodes are connected to VCC (the anode of the + 5 V power supply), and the sleeve is connected to GND (the ground of the +5 V power supply). When the power is connected, a test loop of liquid conductivity is formed between Electrode A and the sleeve as well as Electrode B and the sleeve.

**Figure 2:**
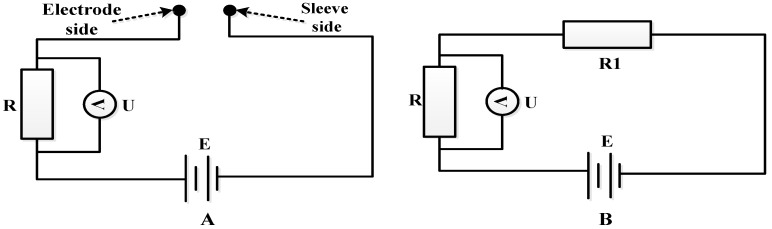
Circuit diagram of the bubble sensor. (**A**) The detection circuit for a single electrode; (**B**) the simplified circuit.

Due to the complexities of the measuring circuit and conductivity computational process, this paper illustrates the detection of bubbles’ relevant parameters using the equivalent circuit shown in Figure 2. The Electrode A and Electrode B detection circuits are identical. The detection circuit for a single electrode is shown in Figure 2A. Figure 2B is the simplified circuit of Figure 2A.

Figure 2 show that when the solution connects the sleeve and the single electrode, a closed circuit is formed because the solution conductivity is not 0, and the voltage *U* between the two ends of the resistance *R* is not 0 (the circuit can be simplified as shown in Figure 2B). Conversely, when bubbles connect the sleeve and the electrode, an open circuit is formed because the gas conductivity is 0 and the voltage *U* between the two ends of the resistance *R* is 0.

### 2.3. Bubble Quantity Detection Principles

When the voltage between the two ends of the resistance R changes from *U* ≠ 0 to *U* = 0, bubbles enter, and when the voltage changes from *U* = 0 to *U* ≠ 0, bubbles leave. Therefore, during the subsequent processing of the output data of the sensor, the bubble quantity can be detected by computing the change in voltage between the two ends of the resistance R.

### 2.4. Two-Phase Flow Pattern Detection Principles 

**Figure 3:**
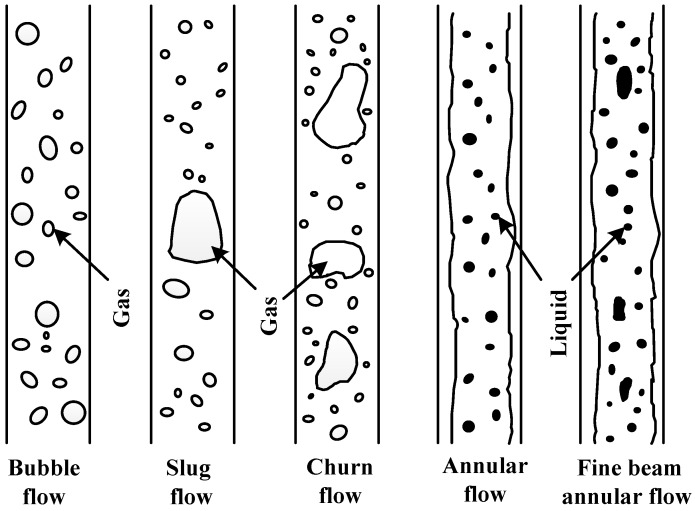
Two-phase flow patterns in vertical CBM wells.

The flow patterns of two-phase flows can be classified according to different inclination angles. This paper studies vertical CBM wells. With an increase in gas content, the flow patterns of two-phase flows can be divided into bubble, slug, churn, annular, and fine-beam annular flows [18], as shown in Figure 3.

**Figure 4:**
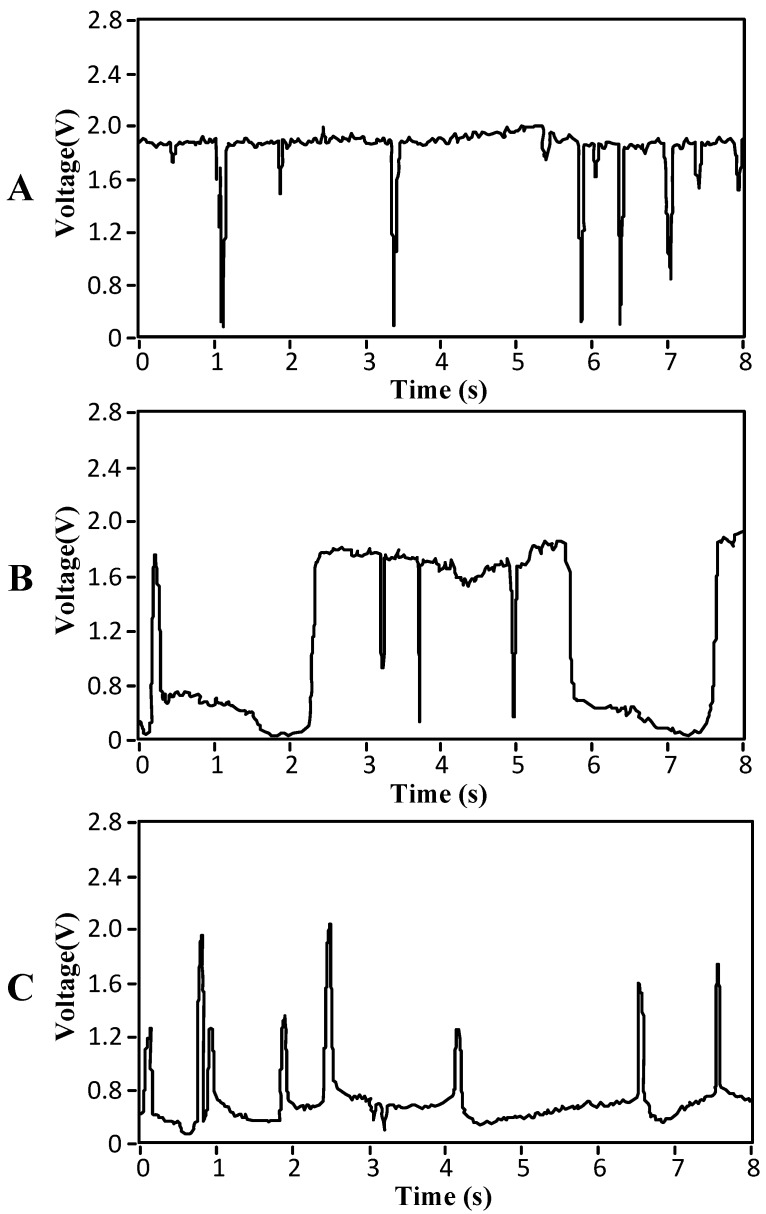
Graph of the two-phase flow pattern. (**A**) Bubble flow; (**B**) slug flow or churn flow; (**C**) annular flow or fine-beam annular flow graph.

The two-phase flow pattern can be judged according to a graph of the collected voltage *U* (as shown in Figure 4). In Figure 4, the abscissa of the curve is time (s), and the ordinate of the curve is the output voltage signal value (V) of the bubble sensor.

The specific flow patterns and judgment methods are as follows. 

Figure 4A shows the measured bubble flow graph. When bubbles pass through the bubble sensor, the output signal of the sensor should be 0. Due to the existence of interference signals, however, the actual output signal is not 0 but is very close to 0.

Figure 4A shows that many small bubbles passed through the sensor during the time period. The features of the flow graph accord with those of bubble flow, and the flow therefore belongs to bubble flow.

Figure 4B shows the measured slug flow graph or churn flow graph. Figure 4B shows that gas and liquid alternately passed through the sensor during the time period. Because the liquids contained bubbles with small diameters, the features of the flow graph accord with those of slug or churn flows.

Because the features of slug flow are similar to those of churn flow, they can be classified as the same kind in actual detection.

Figure 4C shows the measured annular flow graph or fine beam annular flow graph. Figure 4C shows that more gas and less liquid alternately passed through the sensor during the time period (or it can be understood that the gas contained liquid balls). The features of the flow graph accord with those of annular or fine-beam annular flows. 

Because the features of annular flows are similar to those of fine-beam annular flows, they can be classified as the same kind in actual detection.

### 2.5. Velocity Detection Principles

**Figure 5:**
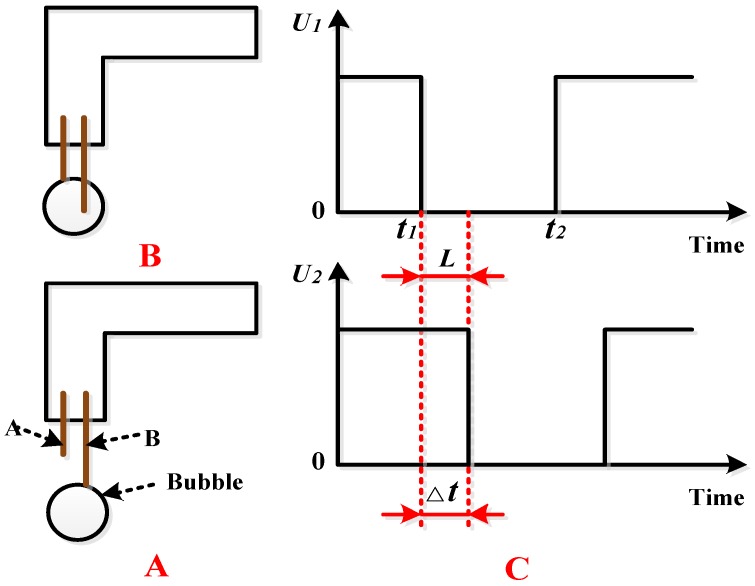
The principle of bubble velocity detection. (**A**) The rising bubble first hit Electrode B; (**B**) the same bubble then hit Electrode A; (**C**) the output signals of this progress.

The detection of bubble velocity needs to be analyzed based on the detection data of Electrode A and Electrode B. The velocity detection principle is shown in Figure 5. 

As shown in Figure 5A, the rising bubbles first hit Electrode B. Because the conductivity of the bubbles is 0, the circuit output voltage *U*_1_ of Electrode B changes from *U*_1_ ≠ 0 to *U*_1_ = 0. Subsequently, bubbles continue to rise, during which process the output voltage *U*_1_ of Electrode B remains 0. The circuit output voltage *U*_1_ of Electrode B changes from *U*_1_ = 0 to *U*_1_ ≠ 0 only when the bubbles move away from Electrode B. 

As shown in Figure 5B, when the bubbles continue to rise and touch Electrode A, the circuit output voltage *U*_2_ of Electrode A changes from *U*_2_ ≠ 0 to *U*_2_ = 0. The bubbles then continue to rise, during which process the output voltage *U*_2_ of Electrode A remains 0. The circuit output voltage *U*_2_ of Electrode A changes from *U*_2_ = 0 to *U*_2_ ≠ 0 only when the bubbles move away from Electrode A. 

In this process, the output signals of Electrode B and Electrode A change with time theoretically, as shown in Figure 5C, where the abscissa is time and the coordinate is the voltage value of the output signal.

*U*_1_ and *U*_2_ are, respectively, the output voltages of Electrode A and Electrode B. The distance *L* between the ends of Electrode A and Electrode B (as shown in Figure 1) is a fixed value, and △*t* in Figure 5C can be calculated according to the sampling frequency of the circuit; therefore, the rising velocity of bubbles is

(2) $$v=\frac{L}{\Delta t}$$

where *v* is the rising velocity of the bubbles, *L* is the distance between the ends of Electrode A and Electrode B, and △*t* is the time required for the bubbles to pass *L*.

### 2.6. Volume Detection Principles

The bubble volume can be detected based on the output data of a single electrode. As an example, consider the output data for Electrode B shown in Figure 5C. Suppose that the circuit output voltage *U*_1_ of Electrode B in the process remains 0 for *t*_1_*t*_2_. The value of *t*_1_*t*_2_ can be calculated according to the sampling frequency of the circuit, and the diameter *d* of the bubble therefore is (assuming that the diametric plane of the spherical bubble passes through Electrode B)

(3) $$d=v\cdot t_{1}t_{2}$$

where *d* is the bubble diameter, *v* is the rising velocity of the bubbles, and *t*_1_*t*_2_ = *t*_2_ − *t*_1_.

We can determine the diameter of the bubble twice because there are two electrodes. Therefore, the final diameter is the average of the two diameters.

In Equation (3), it is assumed that the bubble is a planar circle, that is, the diametric plane of the spherical bubble passes through the electrode. However, this is not the case in actual work, and a calculation error occurs but is still within an admissible range.

Assuming that the bubble is spherical, the bubble volume *S* is

(4) $$S=\frac{4}{3}\pi {\left(\frac{d}{2}\right)}^{3}=\frac{\pi {\left(v\cdot t_{1}t_{2}\right)}^{3}}{6}$$

where *S* is the bubble volume.

## 3. Design of Bubble Sensor

To satisfy the requirements of the detection principles mentioned above, a special sensor for detecting bubbles should be designed in detail prior to manufacturing.

The parameters involved in the design process of the bubble sensor include structural dimension parameters (e.g., the gap *H* between Electrode A and Electrode B, distance *L* between the ends of Electrode A and Electrode B, and the external diameter *D* of the sleeve, as shown in Figure 1) and circuit parameters (the resistance *R*, as shown in Figure 2).

### 3.1. Determination of Structural Parameters

It can be seen from the detection principles of the bubble velocity and volume that the gap *H* between Electrode A and Electrode B, distance *L* between the ends of Electrode A and Electrode B, and external diameter *D* of the sleeve as shown in Figure 1 are of great importance.

If *H* is too large, it is difficult to guarantee that the signals detected by Electrode A and Electrode B come from the same bubble in the velocity detection, which will easily cause a data error in the velocity detection. Therefore, *H* should be as small as possible, assuming that Electrode A and Electrode B are insulated.

If *L* is too large, it is also difficult to guarantee that the signals detected by Electrode A and Electrode B come from the same bubble in the velocity detection, which will easily cause a data error in the velocity detection. When *L* is too small, the time interval for a bubble to pass from Electrode A to Electrode B is too small, that is, △*t* in Figure 5C is too small. In this case, the calculation error will be increased if the sampling frequency of the circuit is used to calculate △*t*, which will finally lead to a larger error in the velocity calculation. Thus, the optimal value of *L* should be obtained.

It is relatively simple to determine the value of *D*. The value of *D* should not be too large; otherwise it will exert a large influence on the original form of the bubble. The value of *D* should be sufficiently small so that Electrode A and Electrode B can be inserted into the sleeve smoothly to guarantee that Electrode A and Electrode B are insulated from the sleeve. 

From a literature review and numerous trials [19,20,21,22,23], it was determined that *H* = 0.3 mm, *L* = 3 mm, and *D* = 1.5 mm.

### 3.2. Determination of Circuit Parameters

It can be determined from Figure 2B that the voltage *U* between the two ends of the resistance *R* is

(5) $$U=\frac{E}{R+R_{1}}R$$

where *E* is the output voltage of the power supply, and *R*_1_ is the equivalent resistance between the electrode and sleeve.

In the actual signal detection, we needed to improve the signal-to-noise ratio of the sensor signal. However, because the noise signal could not be further reduced, we were only able to improve the value of *U* to obtain an optimal signal-to-noise ratio. It can be seen from Equation (5) that the value of *U* increases as the value of the resistance *R* increases. However, because the output power *P* of the power supply could be increased to infinity and the output voltage of the power supply remained the same, it was impossible to increase the value of the resistance *R* to infinity. 

Assuming that the maximum resistance of *R* is *R*_max_, and at this point the current is *I*_min_, the functional relation is

(6) $$P=I_{min}^{2}\left(R_{1}+R_{\max}\right),\text{ and}$$

(7) $$I_{min}=\frac{E}{R_{1}+R_{\max}}$$

where *P* is the output power of the power supply, *E* is the output voltage of the power supply, *I*_min_ is the current passing through the circuit when the resistance *R* is a maximum, *R*_max_ is the maximum resistance *R*, and *R*_1_ is the equivalent resistance between the electrode and sleeve. 

The following equation can be deduced from Equations (6) and (7):

(8) $$R_{\max}=\frac{E^{2}}{P}-R_{1}$$

Therefore, the maximum resistance *R* can be obtained from Equation (8). When the resistance *R* is a maximum, the voltage between the two ends of the resistance *R* is greatest. Assuming that the maximum voltage is *U*_max_, then

(9) $$U_{\max}=E-\frac{PR_{1}}{E}$$

When making a bubble sensor, it is better to increase the precision of the resistance in series with the circuit, but it cannot exceed *R*_max_. When making a data acquisition circuit, the high-level maximum voltage that the circuit can recognize is *U*_max_. 

**Figure 6:**
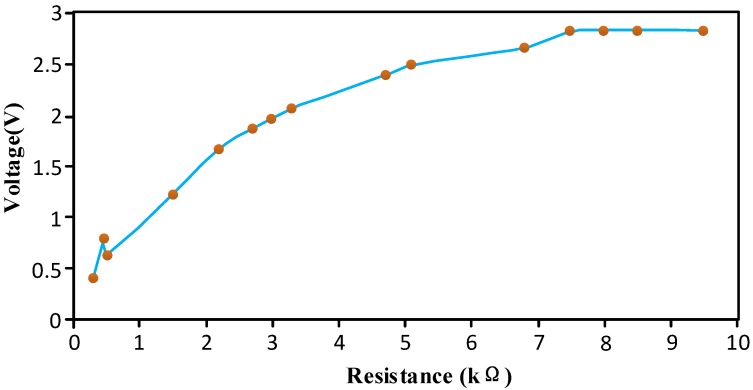
The experimental results of the relationship between the output voltage and resistance of the bubble sensor.

To verify the correctness of the above equation, a test was performed, and Equation (9) was corrected based on the test results. During the test, the circuit resistance changed and the other conditions remained unchanged; the curve of the test results is shown in Figure 6, in which the abscissa is the resistance value and the ordinate is the output voltage value of the bubble sensor. 

Figure 6 shows that when the resistance was between 0 kΩ and 7.5 kΩ, the resistance was positively correlated and nearly in direct proportion with the output voltage. When the resistance was increased above 7.5 kΩ, the output voltage value no longer rose with the increase in resistance. 

For the power supply used in the test, *E* = 5 V and *P* = 2.5 W. It can be deduced from Equation (8) that the maximum resistance was *R*_max_ = 10 − *R*_1_. In addition, it can be deduced that *R*_1_ was approximately 2.5 kΩ with the aid of Figure 6. 

According to Equation (9), the maximum output voltage *U*_max_ of the circuit was 3.75 V, and the actual maximum output voltage of the circuit was 2.828 V. For different resistances, the proportionality coefficient between the actual output voltage and the theoretical output voltage was almost the same, and Equation (9) is therefore correct. 

A proportional relationship exists between the theoretical value and the practical value calculated by Equation (9) due to various factors that include the experimental conditions, temperature, and the data acquisition device. Hence, when making a bubble sensor, Equation (9) can be corrected by virtue of the finished sensor and experimental data. The maximum output voltage after correction is

(10) $$U_{\max}=k\left(E-\frac{PR_{1}}{E}\right)$$

where *k* is the correction coefficient.

For the bubble sensor design reported in this paper, a larger resistance *R* is not necessarily better (*R* ≤ *R*_max_). The power consumption and heat output will increase as *R* increases. Therefore, taking various factors into account, *R* = 3 kΩ was selected for the bubble sensor designed in this study.

### 3.3. Selection of Electrode Materials

The inherent nature of the material causes a “water film phenomenon” between the electrode and sleeve. This phenomenon will cause the measurement accuracy of the bubble sensor to be reduced or the sensor to even fail, and it should therefore be eliminated or minimized. The exiting electrodes use a hydrophobic material coating on the surface of the electrodes, and only a very small region exposed near the tip serves as an electrical contact with the conducting fluid. However, the water film phenomenon persists. Therefore, further research on the electrical material used in the special environments of CBM wells is needed.

#### 3.3.1. Water Film Phenomenon 

**Figure 7:**
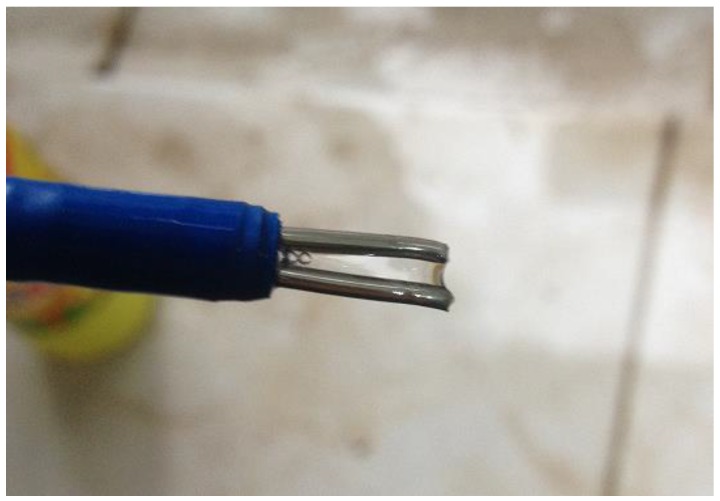
Water film phenomenon.

In practical application, the surface tension and surface free energy between the solution and electrode led to fact that when bubbles touched the bubble sensor, the solution had not fully gotten away from the electrode surface, but had formed a water film between the two electrodes (i.e., between the sleeve and electrode). This is the so-called water film phenomenon that is shown in Figure 7.

In theory, when bubbles pass through the bubble sensor, the sensor detection circuit is open, and its output voltage is 0; when the solution passes through the bubble sensor, the detection circuit of the sensor is closed, and its output voltage is not zero.

However, when the water film phenomenon occurs in the bubble sensor, the water film connects the electrode and sleeve. At that time, although the bubbles have entered the sensor, the sensor is still in a half-conducting state, and the output voltage is not zero. Hence, the water film phenomenon will exert a large influence on the detection accuracy of the bubble sensor.

Because the water film phenomenon is caused by the nature of the material itself, very few materials can avoid this phenomenon. Thus, it can only be minimized as much as possible but not eliminated either theoretically or practically.

#### 3.3.2. Causes of the Water Film Phenomenon and Primary Selection of the Copper Electrode

In essence, the water film phenomenon is caused by the wettability of materials. Thus, the proper electrode materials were selected by analyzing the wettability of materials. Wettability refers to the process of replacing a fluid with another fluid superficially; thus, wettability necessarily involves three phases, and at least two phases are fluid.

The wetting process is divided into three stages: bedew, soaking, and spreading [24]. Bedew refers to the formation of a solid–liquid interface after the solid phase contacts the liquid phase. Soaking refers to the formation of a solid–liquid interface after the solid phase invades the liquid phase. Spreading refers to the formation of a solid–liquid interface after the liquid phase spreads over the surface of the solid phase. 

For the bubble sensor, wetting refers to the process of replacing the liquid phase on the surface of the sensor electrode (solid phase) with a gas phase. There are two kinds of wetting phenomena of the bubble sensor in the wellbore.

1. If there is no wetting between the two-phase flow in the wellbore and the bubble sensor electrode or the wetting process remains bedew, the solution can quickly move away from the electrode when the solution and bubbles alternatively pass through the electrode, so that the water film phenomenon will not occur in most cases. Thus, the measurement results will not be influenced.
2. If the wetting process between the two-phase flow in the wellbore and the bubble sensor electrode remains soaking or spreading, the water film phenomenon will easily occur. When the solution and bubbles alternatively pass through the electrode, the solution fails to quickly move away from the electrode. At this point, if the water film between the two adjacent electrodes adheres, the bubble sensor will fail to judge correctly, finally causing an error data output of the bubble sensor.

Therefore, nonwetting electrode materials should be given preference. The relevant literature shows that metals and their oxides belong to solids with high surface energy and are easily wetted [25,26]. Thus, metals are more easily wetted than nonmetals. Under normal temperatures and pressures, almost all nonmetallic solids are nonconductive. A few conductive nonmetallic solids were also not considered, due to, for example, their low stiffness and inaccessibility. As a result, electrode materials were selected from metals and their oxides.

Specific surface free energy refers to the free energy on a specific surface. The value of specific surface free energy is the same as that of surface tension, and they can be substituted for each other. They are only conceptually different. Hence, the surface free energies of various metals can be compared through the comparison of their surface tensions. The surface tensions of some metals are shown in Table 1 [27].

Table 1 shows that the material with the smallest surface tension is gold. However, gold is unfavorable due to its cost and poor rigidity, and the surface tension of copper (Cu) is second to that of gold. In theory, if copper is selected as the sensor electrode material, data errors can be minimized to the greatest extent, and copper therefore was primarily selected as the electrode material. 

#### 3.3.3. Selection of Stainless Steel Electrodes

Large numbers of laboratory tests using copper as the electrode material have been performed, and the test results show that bubble sensors made from copper will fail to work after immersion in solutions for some time. The sensor cannot detect bubbles but will resume working after its electrode is polished using sand paper.

After verification and analysis, it was found that the above phenomenon is caused by the oxidation reaction of copper in the solution, and the chemical equation is

2Cu + O_2_ = 2CuO (11)

2CuO + CO_2_ + H_2_O = Cu_2_(OH)_2_CO_3_ (12)

If CuO (copper oxide) is generated on the surface of the electrode, Cu (copper) cannot be selected as the electrode material because CuO is nonconductive under its normal state.

If Cu_2_(OH)_2_CO_3_ (basic copper carbonate) is generated on the surface of the electrode, Cu (copper) cannot be selected as the electrode material because Cu_2_(OH)_2_CO_3_ is an ionic compound and is nonconductive in its normal state.

It can be deduced from Table 1 that zirconium and titanium should be sequentially selected as the electrode material if copper fails. However, zirconium and titanium are expensive and difficult to acquire and process, and they therefore are also not favored.

The surface tension of steel (stainless steel) is similar to that of copper, and laboratory tests show that the measurement results of stainless steel electrodes are within the range of the allowable errors. Compared with copper, stainless steel has the following advantages.

1. The nature of stainless steel is relatively stable in moist air and water, and therefore it is not easy for stainless steel to chemically react, whereas copper easily reacts biochemically.
2. The high rigidity of stainless steel ensures that stainless steel electrodes will not be deformed under the impact of two-phase flows in wellbores. On the contrary, the stiffness of copper (or its alloys) is low, and copper therefore is easily deformed by external shocks.

Therefore, stainless steel was finally selected as the electrode material.

## 4. Laboratory and Field Tests

Laboratory and field tests were employed. The measurement range, accuracy, and reliability of the bubble sensor were verified and calculated using laboratory tests. The adaptability of the designed bubble sensor to the environment in CBM wells and its reliability for long-term work was subjected to verification through field tests.

### 4.1. Laboratory Tests

#### 4.1.1. Test Devices

**Figure 8:**
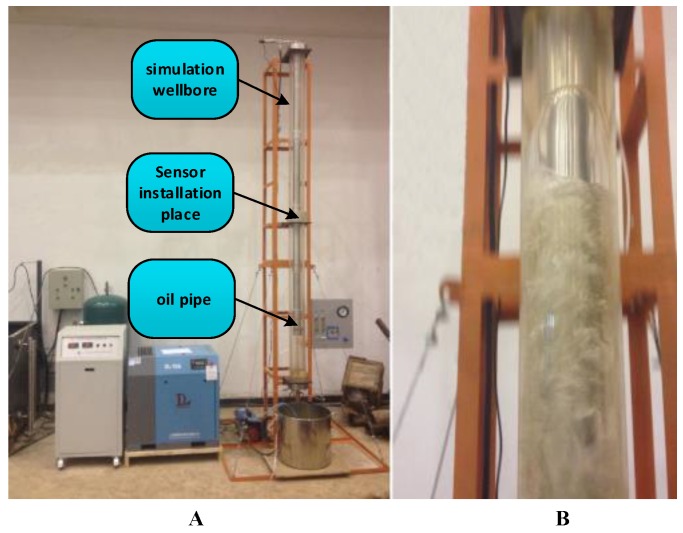
Two-phase flow simulator. (**A**) The two-phase flow simulator; (**B**) The slug flow.

A custom-made two-phase flow simulator that can simulate different flow patterns of two-phase flows was adopted for the laboratory tests. In addition, the simulator was also equipped with a bubble measurement system that can measure bubble velocity and volume and the quantity, pressure, temperature and other parameters of two-phase flows in real time. The two-phase flow simulator is shown in Figure 8. Figure 8A shows the two-phase flow simulator, and Figure 8B shows slug flow, which can be simulated by the two-phase flow simulator.

The size about the two-phase flow simulator is as follows. 

Simulation wellbore: external diameter 145 mm, inner diameter 125 mm, height 3500 mm.

Oil pipe: external diameter 73 mm, inner diameter 62 mm, height 3000 mm.

Installation size: 1500 mm × 1500 mm × 4200 mm. 

#### 4.1.2. Test Process

1. Install the designed bubble sensor on the two-phase flow simulator.
2. After turning on the main power of the two-phase flow simulator, the simulator starts to work.
3. Adjust the pressure and temperature of the simulator to normal pressure and room temperature.
4. Adjust the two-phase flow quantity and gas-phase flow quantity.
5. Because there are more bubbles in the bubble flow, maintain a bubble flow pattern to obtain more test data.
6. Turn on the power supply of the data acquisition system of the simulator. The data acquisition system can acquire and store the velocity and volume of bubbles, and the data acquired by this system are designated standard data.
7. Turn on the power supply of the data acquisition system of the designed bubble sensor. The data acquisition system can acquire and store the velocity and volume of bubbles, and the data acquired by this system are designated acquired data.
8. Constantly adjust the velocity and size of the bubbles in the simulator, and turn off the power supplies of all systems after collecting data for some time.

#### 4.1.3. Test Results

A comparison of the test results and the normal data of the custom-made test device is shown in Table 2. The error was calculated as

(13) $$e_{r}=\frac{A_{d}-S_{d}}{S_{d}}\times 100\%$$

where *e*_r_ is the error, *A*_d_ is the acquired data, and *S*_d_ is the standard data.

Based on numerous experiments and an analysis of Table 2, the following conclusions were obtained.

1. The bubble sensor can detect the flow patterns of two-phase flows.
2. The bubble sensor can detect bubbles with diameters above 2 mm (the volume is calculated by the diameter) and velocities below 0.6 m/s.
3. The bubble sensor’s bubble velocity measurement error was controlled within ±5%, and the measurement error of the bubble volume was controlled within ±7%.

After analyzing the data errors shown in Table 2, the following conclusion was obtained. The velocity measurement error was caused by the detector itself, and Equation (4) for calculating the bubble volume shows that the volume measurement error was caused by five factors:

1. the measurement error of the detector itself;
2. the velocity measurement error accumulated into the volume measurement error after the calculation;
3. When the diameter of the bubble was less than approximately 5 mm, the shape of the bubble was nearly spherical. When the diameter of the bubble was between approximately 5 mm and 9 mm, the shape of the bubble was nearly ellipsoidal. When the diameter of the bubble exceeded 9 mm, the shape of the bubble was nearly that of an irregular ellipsoid [28]. The volume was calculated assuming that the shape of the bubble was spherical, which introduced errors when measuring the volumes.
4. The diameter measurement assumed that the diametric plane of a spherical bubble passed through an electrode, which was impossible in fact, and the measured diameters therefore may have been smaller than their true values.
5. The trajectory was slightly helical rather than rising vertically when a bubble was rising [29], so that the velocity direction and axis of the diameter all slightly changed. As a consequence, the measured velocity and diameter will cause an error.

**Figure 9:**
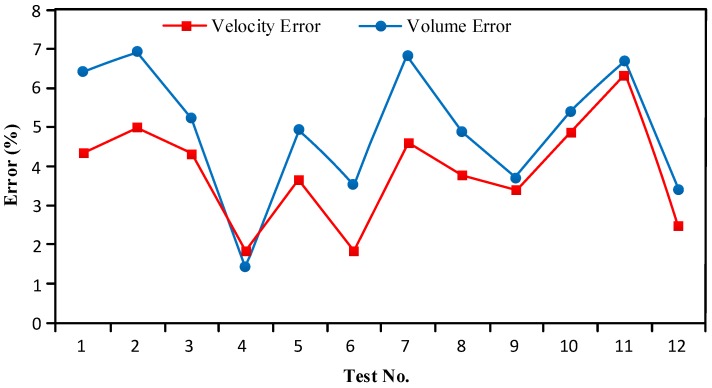
Relationship between the velocity measurement and volume measurement errors.

Figure 9 shows the relationship between the velocity measurement and volume measurement errors. It can be seen from Figure 9 that the trends in the volume measurement and velocity measurement errors are similar, i.e., the volume measurement error increased/decreased as the velocity measurement error increased/decreased. Moreover, the velocity measurement error of a bubble was generally smaller than its volume measurement error, thus proving that the volume error was partially caused by the velocity error. 

### 4.2. Field Tests

The adaptability of the designed bubble sensor to the environment in CBM wells and its reliability for long-term work was verified using field tests.

**Figure 10:**
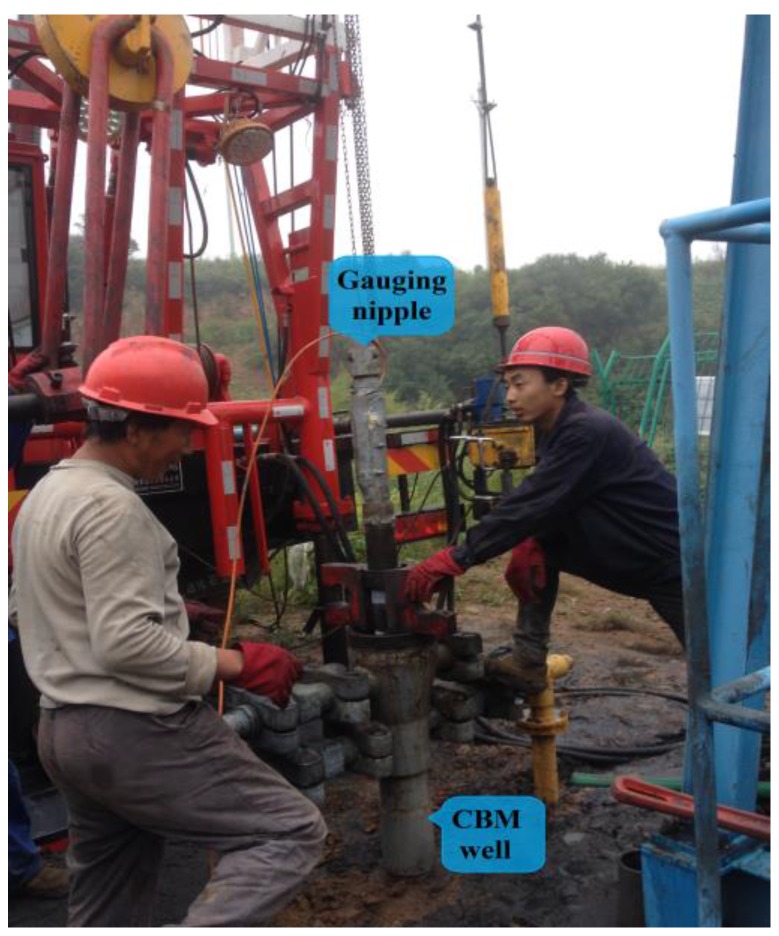
Field of the gauging nipple entering the well.

Test method: Connect the designed bubble sensor to the specially-made gauging nipple and place the gauging nipple into the CBM well with the extraction tube. The data collected by the sensor were transmitted to the ground terminal through cables in real time for display and storage. The field of the gauging nipple entering the well is shown in Figure 10. 

Test location: JS-064 Well, Lanyan CBM Co., Ltd., Jincheng City, Shanxi Province, China.

Test time: On 11 September 2015, the power supply was turned on to collect data. On 28 June 2016, the power supply was turned off to stop the data collection. 

Test environment: In almost nine months of uninterrupted tests, the temperature of the well bottom changed from 5 °C to 50 °C, and the pressure of the well bottom changed from 0 MPa to 10 MPa.

Sample frequency: As data under a CBM well are transmitted to the surface by a cable, the sample frequency must take into consideration transmission line theory and the test. After a number of tests, we determined the sample frequency, which was 400 Hz, and the bubbles’ rise velocity in the CBM well was within the collection range of that transmission rate [28].

Data Storage: The data were stored on an SD card, which had a maximum capacity of 8 GB. 

**Figure 11:**
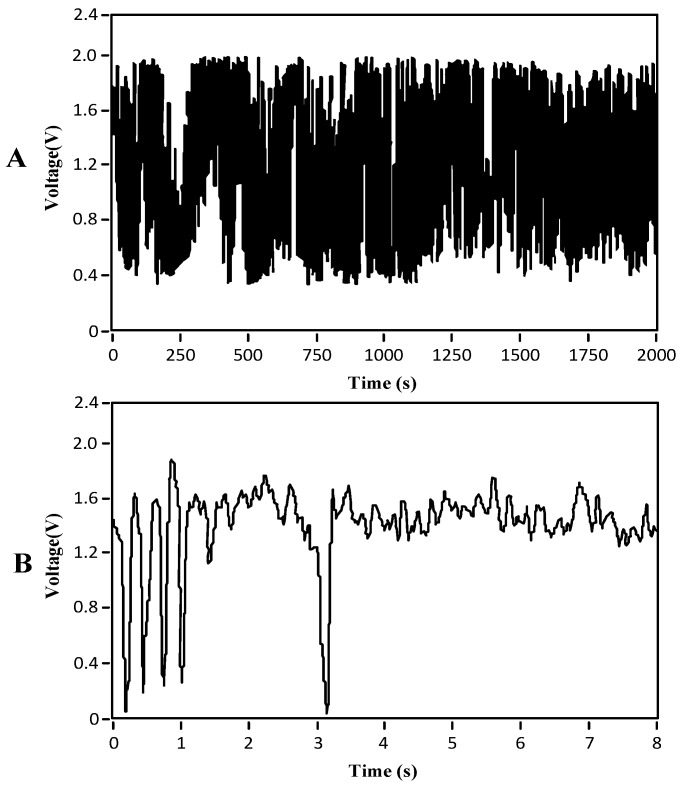
Field test data curve of the bubbles during a certain time period. (**A**) 2000 s of field test data; (**B**) part data for further analysis.

Test conclusion: Almost nine months of uninterrupted data collection (the curve of the collected bubbles during a certain period of time on 28 June 2016, is shown in Figure 11) verified the reliability of the designed bubble sensor.

#### 4.2.1. Flow Pattern

In Figure 11, the plot’s abscissa is time (s), and the ordinate is the value of the output voltage signal of the bubble sensor (V). Figure 11A shows 2000 s of field test data taken on the working dates. Because the quantity of data was too great to discern, we decided to investigate the smaller quantity of data shown in Figure 11B.

The curve in Figure 11B shows that the detected small bubbles were primarily bubble flow, consistent with the field situation, verifying that the designed bubble sensor can be adapted to the environment in CBM wells and is reliable for long-term work.

#### 4.2.2. Bubble Velocity and Volume

**Figure 12:**
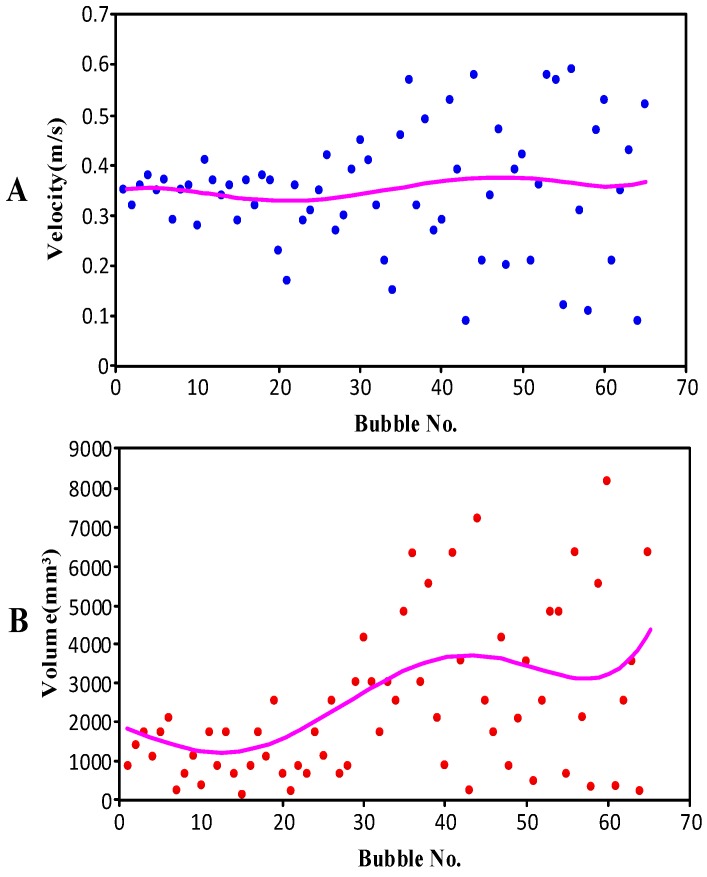
Velocity and volume scatter diagrams for the same group of bubbles. (**A**) The velocity scatter diagram; (**B**) the volume scatter diagram.

Figure 12 shows the velocity and volume scatter diagrams for the same group of bubbles and the line in the figure is the trend line. Figure 12A shows the velocity scatter diagram of a group of bubbles; the abscissa of the scatter diagram is the number of bubbles, and the ordinate of the scatter diagram is the velocity of the bubbles (m/s). Figure 12B shows the volume scatter diagram of the same group of bubbles; the abscissa of the scatter diagram is the number of bubbles, and the ordinate of the scatter diagram is the volume of the bubbles (mm^3^).

In Figure 12A, the velocity of the bubbles is concentrated when the bubble number is within 30, but then it begins to diffuse quickly. In addition, the velocity is the same as the volume (as shown in Figure 12B) for the same group of bubbles. That is to say, the bubbles’ velocities and volumes began to diffuse quickly after bubble number 30. We reached the following conclusions from this phenomenon.

1. The phenomenon was caused by the mutation of the two-phase flow. It may have been the pressure mutations of the coal seam, coalbed water increase, or other geological cause. In short, the condition of the down hole was unstable, and it is therefore better not to adjust the gas drainage and extraction processes during this period.
2. The range of the velocity and volume get bigger, and the average velocity and average volume have an increasing trend.

### 4.3. Bubble Sensor Technical Indicators 

Through the laboratory and field tests, the designed bubble sensor’s technical indicators were finally obtained, as shown in Table 3. 

## 5. Conclusions

Because existing bubble detectors fail to meet the detection requirements in CBM wells, a new bubble sensor has been designed for measuring the flow patterns of two-phase flows, as well as the quantity, velocity, and volume of bubbles in CBM wells based on theoretical research of the conductivity detection principles of two-phase flow bubbles in CBM wells. Related laboratory and field tests proved that the designed sensor has high reliability and can detect the flow patterns of two-phase flows as well as the velocities and volumes of bubbles, with a velocity measurement error of ±5% and a volume measurement error of ±7%. The bubble sensor has the following features when compared with traditional bubble measuring devices applied in ground environments.

(1) Traditional ground measuring devices are inapplicable under the working conditions of CBM wells because of limitations in their measurement principles, installation size, installation accuracy, sealing conditions, and other factors. However, the designed bubble sensor can meet the detection requirements for CBM wells.

(2) The bubble velocity measurement errors of both the traditional ground measuring devices and the designed bubble sensor are basically within ±5%, and their bubble velocity measurement errors therefore are the same [30,31,32]. The bubble volume measurement error of the designed bubble sensor is ±7%, which is slightly larger than that of traditional ground measuring devices [33,34], but the designed bubble sensor nevertheless can meet the requirements of field use. 

(3) The designed bubble sensor can detect two single bubbles in succession and can barely distinguish adhesive bubbles, just like traditional ground measuring devices.

(4) The sensor is designed to measure bubbles that are spherical or ellipsoidal. Hence, the sensor is suitable for all flow patterns but is more suitable for bubble flows.

## Figures and Tables

**Table 1:** Surface tensions of some metals.

Material	Surface Tension /(mN/m)
Gold (Au)	1105
Copper (Cu)	1265
Zirconium (Zr)	1480
Titanium (Ti)	1650
Steel (Fe)	1780
Iron (Fe)	1780
Platinum (Pt)	1865
Palladium (Pd)	1865
Nickel (Ni)	1924
Cobalt (Co)	1936

**Table 2:** Comparison of the test results and normal data.

Test No.	Acquired Data		Standard Data		Error		Flow Pattern
	Velocity/m/s	Volume /mm^3^	Velocity /m/s	Volume /mm^3^	Velocity /%	Volume /%	
1	0.162	4.192	0.155	3.924	+4.32	+6.39	Bubble flow
2	0.221	33.536	0.232	35.851	−4.98	−6.90	
3	0.256	113.184	0.267	119.077	−4.30	−5.21	
4	0.281	268.288	0.276	264.533	+1.80	+1.39	
5	0.302	523.599	0.291	497.908	+3.64	+4.91	
6	0.279	905.472	0.284	937.277	−1.79	−3.51	
7	0.307	1437.856	0.293	1339.950	+4.56	+6.81	
8	0.358	2038.17	0.372	2142.21	−3.76	−4.85	
9	0.429	3162.11	0.415	3050.14	+3.37	+3.67	
10	0.449	3958.87	0.472	4184	−4.87	−5.38	
11	0.571	5939.54	0.537	5568.9	+6.33	+6.66	
12	0.558	7469.18	0.572	7729.95	−2.45	−3.37	

**Table 3:** Technical indicators of bubble sensor.

Technical Indicators	Value
Applicable Medium	Gas-liquid two-phase flow
Velocity Measurement Range	≤0.6 m/s
Velocity Measurement Error	±5%
Volume Measurement Range	≥33.5 × 10^−9^ m^3^ (i.e., *D* ≥ 2 mm)
Volume Measurement Error	±7%
Applicable Pressure	0–10 MPa
Applicable Temperature	0–85 °C
